# Achieving Hepatitis C Micro-Elimination in a High-Burden Province of Thailand: The Phetchabun Model

**DOI:** 10.3390/tropicalmed11080215

**Published:** 2026-08-01

**Authors:** Wijitra Phaengkha, Pornjarim Nilyanimit, Nungruthai Suntronwong, Jiratchaya Puenpa, Duong Hoang Huy Le, Saranya Ngamnimit, Pattama Janpathip, Lapasrada Kamput, Kawin Thongnoi, Wanalee Toomta, Phatcha Wohanklong, Jarunlak Saokeaw, Niphapornt Thammajit, Suwannee Rerngleum, Ananyalak Yoisara, Nitiya Srisuk, Jidapa Suwannachat, Anchalee Karagate, Rujipat Wasitthankasem, Yong Poovorawan

**Affiliations:** 1 Nam Nao Hospital, Nam Nao District, Phetchabun 67260, Thailand; dr.zontok12@gmail.com (W.P.); jarunlak1993js@gmail.com (J.S.); 2 Center of Excellence in Clinical Virology, Department of Pediatrics, Faculty of Medicine, Chulalongkorn University, Bangkok 10330, Thailand; mim_bhni@hotmail.com (P.N.); suntronwong.n@gmail.com (N.S.); jiratchaya.ph@chula.ac.th (J.P.); huyldh@pnt.edu.vn (D.H.H.L.); 3 Medical Biochemistry & Molecular Biology Department, Fundamental Sciences and Basic Medical Sciences, Pham Ngoc Thach University of Medicine, Ho Chi Minh 72510, Vietnam; 4 Phetchabun Provincial Public Health Office, Mueang Phetchabun, Phetchabun 67000, Thailand; saranyangamnimit@gmail.com (S.N.); pattamamiw16@gmail.com (P.J.); 5 Phetchabun Hospital, Mueang Phetchabun, Phetchabun 67000, Thailand; lapasrada21@hotmail.com; 6 Buengsamphan Hospital, Buengsamphan, Phetchabun 67160, Thailand; oumbun1@gmail.com; 7 Lomsak Hospital, Lomsak, Phetchabun 67110, Thailand; wanaree8595@gmail.com; 8 Chondaen Hospital, Chondaen, Phetchabun 67150, Thailand; nurse.er2520@gmail.com; 9 Si Thep Hospital, Si Thep, Phetchabun 67170, Thailand; maii_duckling@hotmail.com; 10 Wang Pong Hospital, Wang Pong, Phetchabun 67240, Thailand; suwannee.rlm@gmail.com; 11 Wichianburi Hospital, Wichianburi, Phetchabun 67130, Thailand; noey.np.241@gmail.com; 12 Lomkao Crown Prince Hospital, Lom Kao, Phetchabun 67120, Thailand; aomamnitiyayindee@gmail.com; 13 Nongphai Hospital, Nong Phai, Phetchabun 67140, Thailand; jidapa.suwan19@gmail.com; 14 Khao Kho Hospital, Khao Kho, Phetchabun 67270, Thailand; gunpom19@gmail.com; 15 National Center for Genetic Engineering and Biotechnology, National Science and Technology Development Agency, Thailand Science Park, Khlong Luang, Pathum Thani 12120, Thailand; rujipat.wasitthankasem@gmail.com; 16 The Royal Society of Thailand, Sanam Sueapa, Dusit, Bangkok 10300, Thailand

**Keywords:** hepatitis C, micro-elimination, Phetchabun, Thailand, test-to-treat, sofosbuvir/velpatasvir, rapid diagnostic test, universal health coverage

## Abstract

The World Health Organization targets hepatitis C virus (HCV) elimination as a public-health threat by 2030. Phetchabun province, in lower northern Thailand, has historically reported one of the country’s highest HCV seroprevalence rates. We describe a province-wide, decentralized test-to-treat micro-elimination program implemented within Thailand’s universal health-coverage system. Adults aged 35–64 years (2020–2024) and individuals born before 1992 (late 2023) across all 11 districts were screened using fingerstick anti-HCV rapid diagnostic tests at sub-district health-promoting hospitals. Reactive participants underwent confirmatory HCV-RNA testing at three regional nodes. Eligible patients received pangenotypic sofosbuvir/velpatasvir for 12 weeks, with ribavirin added for cirrhosis. Sustained virological response at 12 weeks post-treatment (SVR12) was the primary outcome. Of 400,567 targeted individuals, 389,547 (97.3%) were screened; 18,340 (4.71%) were anti-HCV reactive. Confirmatory HCV-RNA testing was completed in 14,845 (80.9%), of whom 10,996 (74.1%) had active infection. Of 8961 treatment-eligible patients, 8842 (98.7%) received DAA therapy. Among 6719 evaluable for SVR12, 6474 (96.4%) achieved cure. This simplified, domestically financed, decentralized test-to-treat model achieved WHO-target-level diagnosis, treatment, and cure rates at provincial scale, offering a scalable framework for HCV micro-elimination in low- and middle-income countries.

## 1. Introduction

Viral hepatitis remains a major global public-health problem. According to the World Health Organization (WHO) 2024 Global Hepatitis Report, an estimated 254 million people are living with chronic hepatitis B virus (HBV) infection and 50 million with chronic hepatitis C virus (HCV) infection worldwide, with approximately 1.3 million deaths attributed to viral hepatitis in 2022, comparable to mortality from tuberculosis [1,2]. In 2016, the World Health Assembly endorsed the Global Health Sector Strategy on Viral Hepatitis, which set the goal of eliminating viral hepatitis as a public-health threat by 2030 through a 90% reduction in new chronic HBV and HCV infections, an 80% treatment coverage among diagnosed patients, and a 65% reduction in hepatitis-related mortality, with at least 90% of infected individuals diagnosed [3,4]. Thailand endorsed these targets and integrated them into its National Strategic Plan on Viral Hepatitis; however, early implementation was constrained by limited budget allocation and the high cost of antiviral therapy [5].

In Thailand, viral hepatitis is dominated by chronic HBV and HCV infections [5,6]. For HBV, a highly effective vaccine introduced into the Expanded Program on Immunization (EPI) for all newborns in 1992, together with universal precaution practices and stringent blood-bank screening, has driven a marked decline in HBV prevalence from 6 to 8% before 1992 to 1.68% in 2024, with the carrier rate among children aged < 10 years now below 0.1% [6,7,8]. For HCV, no vaccine is available, but pangenotypic direct-acting antivirals (DAAs) can cure more than 95% of treated patients [9,10,11]. The originator-brand DAA regimens were initially prohibitively expensive in Thailand; the situation changed when generic sofosbuvir-based regimens became available, and in early 2021, the National Health Security Office (NHSO) added pangenotypic sofosbuvir/velpatasvir (SOF/VEL) to the Universal Coverage Scheme benefit package, enabling access without cost to patients [12,13].

Following the 2016 WHO call for elimination, the Centre of Excellence in Clinical Virology, Faculty of Medicine, Chulalongkorn University, prioritized hepatitis C as the most achievable elimination target given the existence of curative therapy, and selected Phetchabun, a province in lower northern Thailand with one of the highest reported HCV seroprevalence rates in the country and a correspondingly high incidence of liver cancer, as a pilot site for a province-wide micro-elimination program [14,15].

Baseline epidemiological work began in Phetchabun in 2015. A randomly sampled cross-sectional study in adults aged 30–64 years compared two high-burden districts in Phetchabun (Lom Sak and Lom Kao, *n* = 1667) with the neighboring Chum Phae district of Khon Kaen province (*n* = 1410). Anti-HCV seroprevalence in Phetchabun was about five-fold higher than in Khon Kaen (15.5% vs. 3.6%) and substantially exceeded the national average of 1–2% reported in earlier nationwide surveys [14,15]. A subsequent study in young adults aged 18–30 years residing in the same Phetchabun districts showed an anti-HCV seroprevalence of <4% in those aged 18–34 years that rose abruptly to ≥15% in those aged ≥ 35 years, identifying individuals born before 1983 as the principal birth cohort harboring chronic HCV infection [16,17]. These data established that DAA-based treatment should be prioritized in this older birth cohort to reduce the long-term burden of cirrhosis and hepatocellular carcinoma (HCC) [17,18].

The Phetchabun “test-to-treat” micro-elimination project was designed in 2018. At that time, the province had a population of approximately 995,000, of whom about 438,000 were aged 35–64 years. A 1:100 province-wide sample of 4753 individuals aged 35–64 years was drawn proportionally from every sub-district and tested for anti-HCV using an automated chemiluminescent microparticle immunoassay (ARCHITECT anti-HCV assay, Abbott Diagnostics, Chicago, IL), with confirmation of active infection by HCV-RNA testing [18]. Anti-HCV seroprevalence was higher in the northern districts and lower in the south. Of the seropositive individuals, 76% had detectable HCV RNA, and more than half of those with active infection were infected with HCV genotype 6, supporting the choice of a pangenotypic regimen [18,19]. To enable point-of-care screening, four commercial rapid diagnostic tests (RDTs) for anti-HCV (Abon, Blue Cross, Healgen, and SD Bioline) were evaluated under field conditions and found to have comparable diagnostic performance, supporting their use as a first-line screening tool [20].

Building on these findings, the province-wide test-to-treat program, the Phetchabun model, was launched in early 2020 [21]. Anti-HCV screening was performed using a fingerstick whole-blood RDT, and reactive samples underwent confirmation by automated qualitative real-time RT-PCR, with confirmation testing operationalized from early 2021 onward [21]. The initial target population was adults aged 35–64 years across all eleven districts. In October 2023 (Thai fiscal year 2024), the Department of Disease Control of the Ministry of Public Health and the NHSO expanded eligibility to include all individuals born before 1992 (i.e., the pre-universal-HBV-vaccination birth cohort), and the Phetchabun program adjusted its target population accordingly [12,22]. NHSO has reimbursed SOF/VEL for HCV treatment under universal health coverage since March 2021 [12,13].

The present report consolidates more than five years of programmatic and research data from the Phetchabun model, summarizing progress in screening, confirmatory testing, treatment uptake, and treatment outcomes. The intent is to document a scalable, domestically financed, decentralized micro-elimination strategy that can serve as a reference for other Thai provinces and for other low- and middle-income countries (LMICs) with comparable HCV epidemiology.

## 2. Materials and Methods

### 2.1. Project Framework and Ethics

The Phetchabun province test-to-treat HCV micro-elimination project was supported by the Grand Challenge Fund of the National Research Council of Thailand (NRCT). The protocol was approved by the Institutional Review Board (IRB) of the Faculty of Medicine, Chulalongkorn University (IRB No. 028/63). Research-funded operations ran from early 2021 to early 2024; thereafter, the test-to-treat workflow continued as a routine service under the Department of Disease Control, Ministry of Public Health, and the NHSO. The present analysis combines primary research data and secondary service data captured under the same operational framework, covering the period from program inception in 2020 through April 2026. Selected interim findings have been published previously [18,20,21].

### 2.2. Study Setting

**Figure 1:**
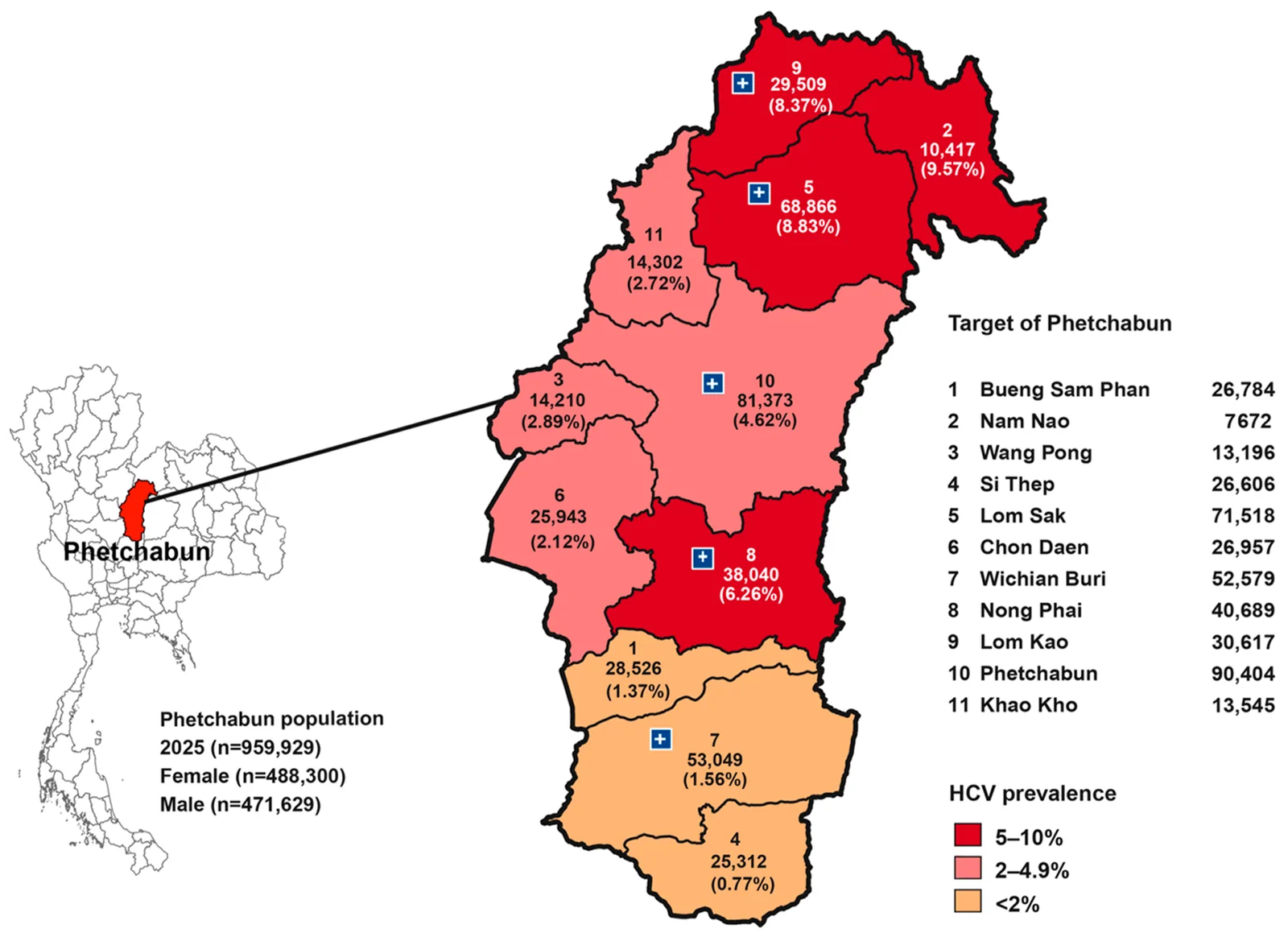
Map of Phetchabun Province and the study districts, showing the total provincial population, the target population, and the results of anti-HCV screening. Administrative boundaries were obtained from the Database of Global Administrative Areas (GADM, version 4.1; https://gadm.org, accessed on 6 June 2026) and processed in R software (version 4.4.2).

Phetchabun is a province in lower northern Thailand, located approximately 350 km north of Bangkok, covering 12,668 km^2^ (Figure 1). The province comprises 11 districts and 117 sub-districts, with a total population of 959,929 (471,629 men and 488,300 women). Health-care delivery is supported by 12 public hospitals, 2 private hospitals, and 155 sub-district health-promoting hospitals. The majority of residents are engaged in agriculture. The provincial map (Figure 1) was generated in R software (version 4.4.2) using the sf and ggplot2 packages, with administrative boundary data obtained from GADM (version 4.1; https://gadm.org (accessed on 6 June 2026)).

### 2.3. Study Period and Target Population

**Figure 2:**
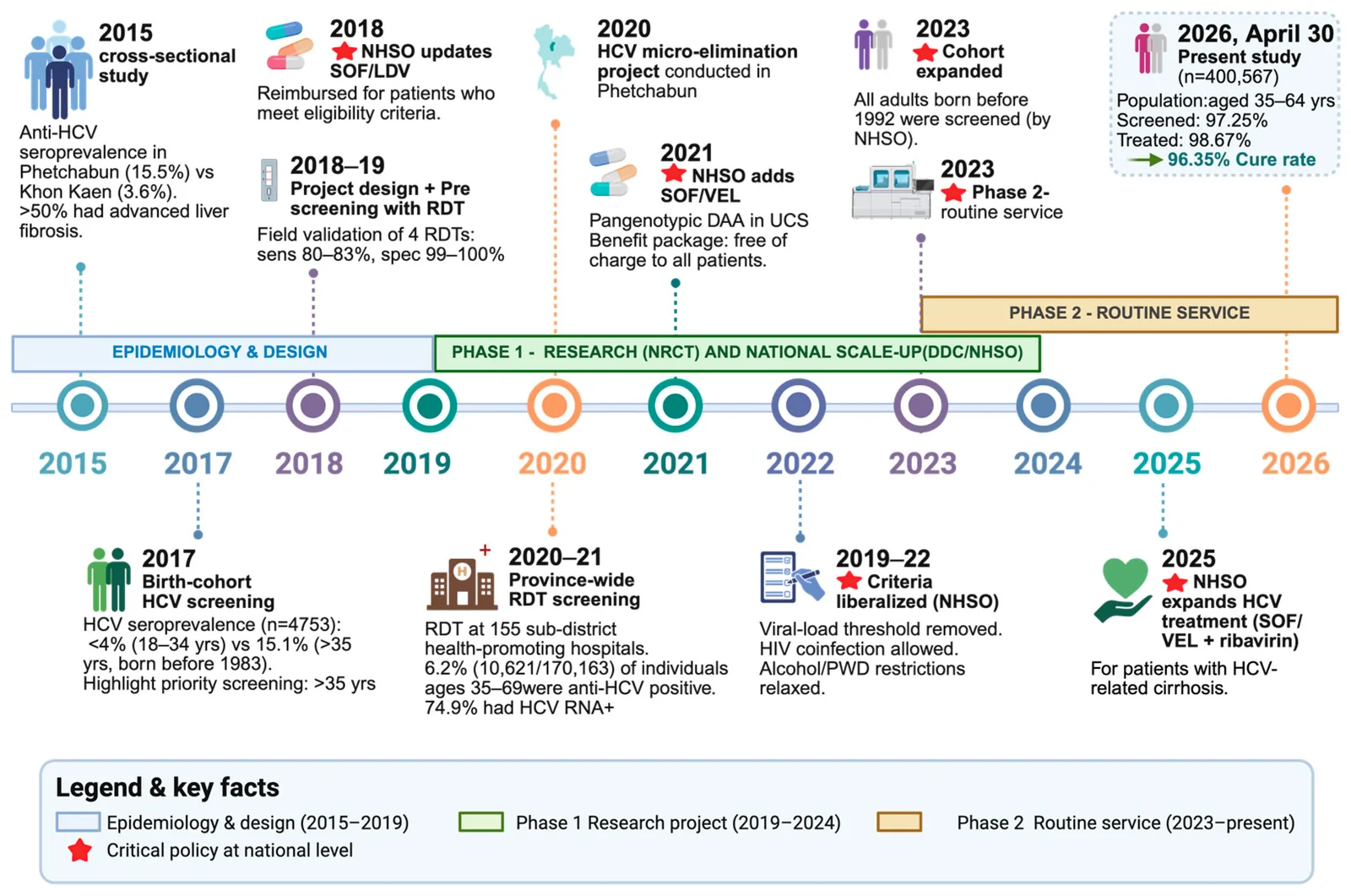
Programmatic and policy timeline of the Phetchabun HCV micro-elimination model, 2015–2026. Created in BioRender. Poovorawan, Y. (2026) https://BioRender.com/t1bou3b (accessed on 6 June 2026).

The program was implemented in two consecutive phases. In Phase 1 (early 2020 to September 2024), conducted under the Grand Challenge research project, point-of-care anti-HCV screening with fingerstick RDTs was paired with qualitative real-time RT-PCR confirmation, targeting adults aged 35–64 years. In Phase 2 (from late 2023 onward), screening was implemented under the policies of the Department of Disease Control and the NHSO; eligibility was broadened to individuals born before 1992 (one-time lifetime screening), with annual rescreening offered to high-risk groups including people living with HIV (PLHIV), people who inject drugs (PWID), men who have sex with men (MSM), incarcerated individuals, and health-care personnel. The present analysis is restricted to the one-time screening cohort, which corresponds to a target population of 400,567 individuals. The timeline of the planning, testing, and treatment of the project, and the NHSO and DDC criteria, are shown in Figure 2.

### 2.4. Diagnostic Procedures

During Phase 1, fingerstick whole-blood anti-HCV screening was performed using the SD Bioline HCV rapid diagnostic test (Standard Diagnostics, Yongin-si, Gyeonggi-do, Korea), which demonstrated 80% sensitivity (95% CI: 70.8–87.3%) and 100% specificity (95% CI: 96.4–100.0%) in laboratory evaluation against a panel of anti-HCV-positive and anti-HCV-negative samples [20]. Reactive samples were forwarded to designated provincial laboratories for confirmation by qualitative HCV-RNA testing on the automated cobas^®^ 4800 system (cobas^®^ HCV test, Roche Diagnostics, Mannheim, Germany), an integrated platform that performs both viral RNA extraction and real-time RT-PCR; 200 μL of plasma was used per assay, with a lower limit of detection of 15.2 IU/mL [21]. During Phase 2 (from October 2024), screening continued to use commercially available, Thai FDA–approved anti-HCV RDTs distributed through the public-health system. Confirmatory testing in Phase 2 used quantitative HCV RNA viral-load assays performed at commercial laboratories accredited by the NHSO, with reimbursement and sample logistics organized at the district level.

All HCV-RNA-positive individuals subsequently underwent additional pre-treatment evaluation, including a complete blood count (CBC) and liver-function tests. Liver fibrosis was assessed using two non-invasive indices: the aspartate aminotransferase-to-platelet ratio index (APRI) and the fibrosis-4 (FIB-4) score [23,24]; no patient in this program underwent liver biopsy.

### 2.5. Treatment Eligibility and Exclusion Criteria

Since late 2021, the NHSO has applied the National List of Essential Medicines criteria for HCV treatment with SOF/VEL [12,13], broadly aligned with international guidance [25,26]. Initially, eligibility required: age 18–70 years; treatment-naïve status, or previous failure or intolerance of pegylated interferon plus ribavirin [25]; HCV-RNA ≥ 5000 IU/mL; significant fibrosis (Metavir stage ≥ F2 on liver biopsy, transient elastography > 7 kPa, FIB-4 > 1.45 or APRI > 0.5), consistent with EASL guidance that non-invasive tests should be used in place of liver biopsy to assess fibrosis severity [23,24,26]; estimated glomerular filtration rate (eGFR) ≥ 30 mL/min [25]; for cirrhotic patients with Child-Pugh score B or C, a Model for End-Stage Liver Disease (MELD) score ≤ 18 was required [25,26]; abstinence from alcohol and illicit substances for ≥6 months [12,13]; for HIV-coinfected patients on highly active antiretroviral therapy (HAART), CD4 count ≥ 200 cells/mm^3^ and HIV viral load < 50 copies/mL [25]; for HBV-coinfected patients, concurrent HBV therapy [25]; and for patients with previous malignancy, ≥6 months of disease-free status [12,13].

Liver biopsy was listed as one option under the NHSO policy criteria but was not performed for any patient in this program; fibrosis staging was assessed exclusively using the non-invasive indices (APRI and FIB-4) described in Section 2.4, consistent with EASL guidance that non-invasive tests should be used in place of liver biopsy [26].

Patients with cirrhosis (assessed by APRI, FIB-4, or clinical judgment) received SOF/VEL plus ribavirin under the supervision of a gastroenterology specialist. Contraindications included pregnancy or unwillingness to use contraception during therapy, uncontrolled severe comorbidity, ongoing chemotherapy, active opportunistic infection in HIV, and decompensated cirrhosis with MELD score > 18. In 2022, the criteria were liberalized: viral-load thresholds were removed (qualitative HCV RNA, quantitative viral load, or HCV core antigen all accepted), restrictions on alcohol and substance use were relaxed, and HIV co-infection was permitted in the absence of active opportunistic infection, particularly tuberculosis. SOF/VEL plus ribavirin remained recommended for cirrhotic patients with a MELD score ≤ 18.

### 2.6. Treatment Discontinuation Criteria

Treatment was completed after 12 weeks of therapy. Therapy was discontinued early in cases of uncontrolled exacerbation of comorbidity, persistent non-adherence to medical advice, or active alcohol consumption during treatment.

### 2.7. Treatment Regimen

HCV-RNA-positive patients received oral SOF/VEL (sofosbuvir 400 mg/velpatasvir 100 mg) once daily, a pangenotypic regimen, dispensed monthly for three consecutive months (total duration 12 weeks). Sustained virological response was assessed 12 weeks after the last dose (SVR12). SOF/VEL has been included in the Thai National List of Essential Medicines for HCV treatment since early 2021 [12,13].

### 2.8. Follow-Up

HCV-RNA-positive patients were initially evaluated by a physician at the district treatment unit. Subsequent follow-up during the 12-week treatment course was conducted by nurse practitioners, with assessment of treatment outcome by HCV-RNA testing 12 weeks after completion (SVR12). Patients who remained HCV-RNA-positive after treatment (treatment failure) were referred to a gastroenterology specialist for re-treatment with SOF/VEL plus ribavirin.

### 2.9. Assessment of Liver Fibrosis

APRI and FIB-4 were used as non-invasive markers to assess liver fibrosis. APRI was categorized as <0.5, 0.5–1.5, and >1.5, while FIB-4 was categorized as <1.45, 1.45–2.67, and >2.67 according to established clinical cut-offs [23,24]. Age-adjusted FIB-4 cut-offs were applied for patients aged ≥ 65 years (<2.0, 2.0–2.67, >2.67). The age-adjusted FIB-4 approach in this study draws on evidence that FIB-4 diagnostic accuracy declines significantly with age in chronic hepatitis C cohorts specifically [27], and follows the age-specific cut-off framework (lower threshold 2.0, upper threshold 2.67 unchanged) validated by McPherson et al. [28].

### 2.10. Data Analysis

Programmatic data were summarized as the cumulative numbers screened (and as a percentage of the target population), the number and percentage of anti-HCV–reactive participants by district, the proportion of reactive participants undergoing confirmatory HCV-RNA testing, the proportion confirmed as HCV-RNA-positive by district, the number entering treatment, the number who died before treatment, and the SVR12 cure rate among those who completed treatment and reached the 12-week post-treatment evaluation.

Baseline demographic and clinical characteristics were compared between patients who achieved SVR12 and those with DAA treatment failure. Categorical variables were compared using the chi-square test or Fisher’s exact test, as appropriate for cell counts; continuous variables (age, BMI) were compared using the Mann–Whitney U test, as these were not normally distributed. A two-sided *p*-value < 0.05 was considered statistically significant. Statistical analyses were performed using Python (version 3.12.3) with the SciPy (version 1.17.1) and pandas (version 3.0.2) libraries.

### 2.11. Generative AI and AI-Assisted Technologies in the Writing Process

Generative AI tools (GPT-4o and Claude Sonnet 4.5) were used exclusively to refine language and improve readability, including translation from Thai to English. All content, concepts, and findings were wholly conceived and developed by the authors. The authors subsequently reviewed the final manuscript to ensure its accuracy, integrity, and adherence to academic standards.

## 3. Results

### 3.1. HCV Screening Coverage

The Phetchabun test-to-treat micro-elimination program, planned from 2018, began screening in 2020 and confirmatory HCV-RNA testing in 2021, initially targeting adults aged 35–64 years and, from late 2023, the broader pre-1992 birth cohort under the joint policy of the Department of Disease Control and the NHSO. The target population for the present analysis comprised 400,567 individuals (out of a total provincial population of approximately 960,000). As of 8 April 2026, cumulative anti-HCV screening using fingerstick RDTs had been performed in 389,547 individuals, equivalent to 97.25% of the target population. Anti-HCV reactivity was detected in 18,340 participants (4.71% of those screened). The seroprevalence was higher in the northern districts of the province (Lom Kao, Lom Sak, Nam Nao) than in the southern districts (Si Thep, Wichian Buri, Bueng Sam Phan); district-level data are presented in Table 1, and the geographical distribution is shown in Figure 1.

### 3.2. Confirmatory HCV-RNA Testing

Of the 18,340 anti-HCV-reactive individuals identified by RDT, 14,845 (80.94%) underwent confirmatory HCV-RNA testing (qualitative or quantitative), of whom 10,996 (74.07% of those tested) were confirmed as actively infected (HCV-RNA positive). District-level results are presented in Table 2.

### 3.3. Linkage to Treatment

Of the 10,996 HCV-RNA-confirmed individuals province-wide, 75 refused the treatment, 70 was lost to follow up, 407 had treatment-limiting conditions, 181 had hepatocellular carcinoma, 339 died before treatment, 339 died before treatment could be initiated and 963 were already in care or being treated outside the Phetchabun network, The remaining 8961 individuals constituted the eligible target population for treatment, of whom 8842 (98.67%, 119 awaiting for treatment) had received DAAs therapy for treatment. District-level details are presented in Table 3.

### 3.4. Treatment Outcomes

**Figure 3:**
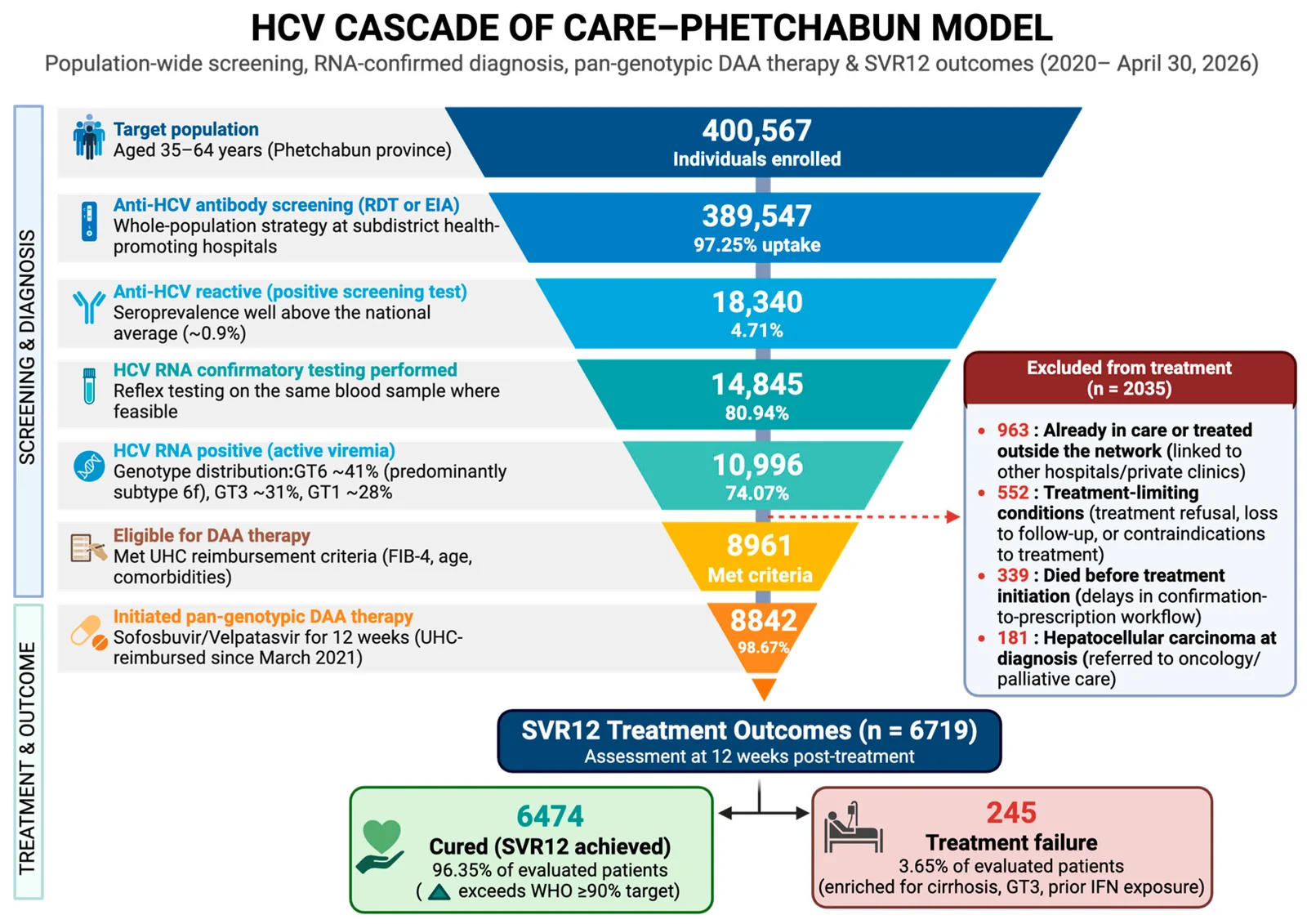
Overview of the study population from screening, confirmatory testing, initiation of direct-acting antiviral (DAA) therapy, and treatment outcomes. The icons represent each stage of the HCV care cascade, including the target population, anti-HCV antibody screening, anti-HCV seropositivity, HCV RNA confirmatory testing, HCV RNA positivity, eligibility for DAA therapy, treatment initiation, SVR12 achievement (cure), and treatment failure. The color scheme distinguishes the screening and diagnostic phase (blue and green), treatment eligibility and treatment initiation (yellow and orange), treatment outcomes (green for SVR12 achieved and pink for treatment failure), and reasons for treatment exclusion (red). Created in BioRender. Poovorawan, Y. (2026) https://BioRender.com/rs31eze (accessed on 6 June 2026).

By April 2026, of the 8961 patients eligible for treatment, 8842 had received DAAs treatment. Among them, 6719 completed the 12-week regimen and underwent SVR12 evaluation. HCV RNA was undetectable in 6474 patients, corresponding to a cure rate of 96.35%, while 245 patients (3.65%) remained HCV-RNA-positive and were classified as treatment failures. The flow of the study population, including screening, confirmatory HCV RNA testing, initiation of direct-acting antiviral (DAA) treatment, and treatment outcomes, is shown in Figure 3.

### 3.5. Factors Associated with DAA Treatment Failure

Compared with patients who achieved SVR, those with treatment failure were more frequently male (89.0% vs. 73.8%, *p* = 0.002), had a higher prevalence of any comorbidity (61.0% vs. 46.0% had at least one condition, *p* = 0.004), and more often had a history of liver cirrhosis (19.2% vs. 6.0%, *p* < 0.001) or injection drug use (8.2% vs. 1.0%, *p* < 0.001) (Table 4). Regular medication adherence was less common among patients with treatment failure (80.8% vs. 95.0%, *p* < 0.001). In contrast, age, BMI, antiviral regimen, and baseline fibrosis severity by APRI or FIB-4 (including age-stratified FIB-4 cut-offs) did not differ significantly between groups (all *p* > 0.05).

### 3.6. Overall Program Performance Against WHO 2030 Targets

**Figure 4:**
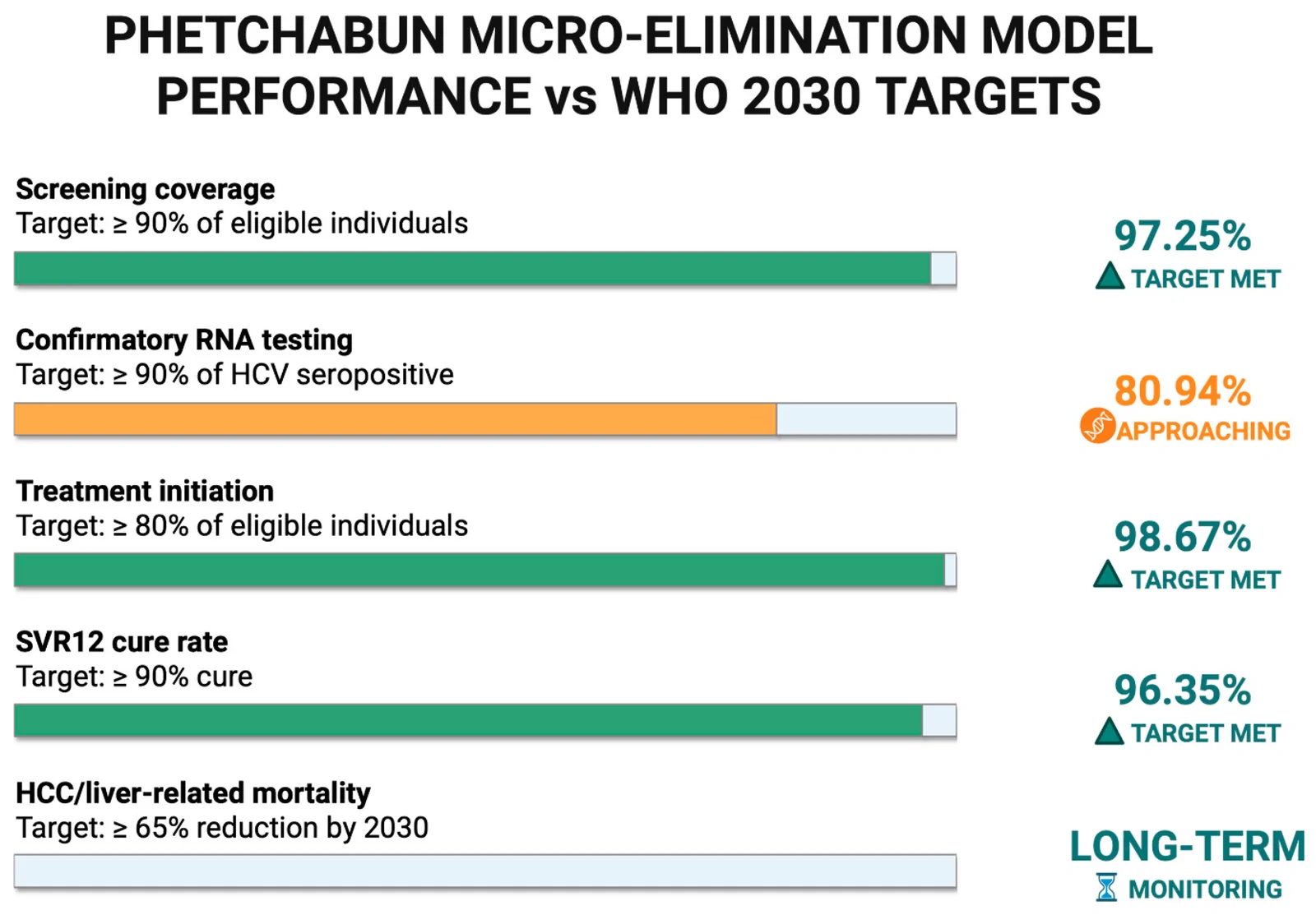
Performance of the Phetchabun HCV micro-elimination model against WHO 2030 elimination targets, 2020 to 8 August 2025. The triangle symbol denotes that the corresponding WHO 2030 target has been achieved (Target Met), the clock symbol indicates that the indicator is Approaching the WHO target, and the hourglass symbol denotes Long-term Monitoring. Green indicates indicators that met the WHO target, orange indicates indicators approaching the target, and gray indicates indicators requiring continued long-term monitoring. Created in BioRender. Poovorawan, Y. (2026) https://BioRender.com/rwg6gw8 (accessed on 6 June 2026).

Overall, the Phetchabun model met or exceeded WHO 2030 elimination targets for screening coverage, treatment initiation, and SVR12 cure rate, and was approaching the target for confirmatory HCV-RNA testing; long-term monitoring of the HCC and liver-related mortality target is ongoing (Figure 4). These results, summarizing the full screening-to-cure cascade described above, indicate that a provincial-scale, decentralized test-to-treat strategy can achieve WHO programmatic benchmarks within a domestically financed universal health coverage system.

## 4. Discussion

The Phetchabun model demonstrates that WHO 2030 hepatitis C elimination targets can be achieved at the provincial scale within a low- or middle-income country (LMIC) through a decentralized test-to-treat strategy embedded in a universal health coverage (UHC) system [3,4,5]. WHO defines elimination as an 80% reduction in new HCV infections and a 65% reduction in HCV-related mortality versus 2015 baselines, with intermediate targets of diagnosing >90% and treating >80% of infected individuals [1,3,4]; only a minority of countries are currently on track to meet these goals [1,29]. Micro-elimination, formalized by Lazarus and colleagues in 2017–2018, breaks national targets into tractable sub-goals focused on defined populations or geographies [30,31]. Phetchabun, with historical sub-district anti-HCV seroprevalence of 7–15% and one of Thailand’s highest provincial liver-cancer rates [5,14,15,18,32,33], was a strategic site for the country’s first geographically targeted micro-elimination project, covering all 11 districts and ~960,000 residents.

From 2020 to April 2026, the program met or exceeded WHO programmatic targets: 389,547 of 400,567 targeted individuals (97.25%) were screened; 18,340 (4.71%) were anti-HCV reactive; 14,845 (80.94%) completed confirmatory HCV-RNA testing, of whom 10,996 (74.07%) had active infection; 8842 of 8961 eligible patients (98.67%) received SOF/VEL; and 6474 of 6719 evaluable patients (96.35%) achieved SVR12. These cure rates are consistent with global real-world SOF/VEL evidence and with >95% SVR reported by other national elimination programs [9,10,11,34,35,36,37,38].

Comparison with other elimination programs situates these findings. National programs achieving high coverage, such as Egypt, Georgia, and Rwanda, share sustained political commitment, low-cost pangenotypic DAA procurement, task-shifting to non-specialist providers, decentralized care, and integrated electronic data systems [29,34,35,36,39,40,41,42,43,44,45,46,47,48]; Phetchabun replicates these delivery principles at provincial rather than national scale, without external donor financing or a national political mandate. By contrast, Pakistan’s experience illustrates how fragmented sub-national governance and limited political continuity constrain population-level impact despite comparable ambition [49,50,51,52]; Phetchabun was designed to avoid these pitfalls by embedding all data within Thailand’s NHSO national system. Population-targeted programs in Iceland, Australia, Spain, the Netherlands, and Scotland further demonstrate that prioritizing settings of concentrated transmission improves cascade outcomes even in low-prevalence, high-income settings [37,53,54,55,56,57,58,59,60,61,62]; while Phetchabun’s epidemiology reflects historical transmission in an older birth cohort rather than active injecting drug use, the same principle of targeted intervention applies. Community-based programs in Taiwan and rural Egypt achieved diagnostic and treatment rates broadly comparable to Phetchabun’s [38,63,64,65]. Relative to these programs, Phetchabun’s distinctive contribution is province-wide coverage integrated directly into Thailand’s UHC reimbursement system and national guidelines, using a low-cost, pangenotypic RDT-to-qualitative-RNA workflow suited to Thailand’s mixed HCV genotype distribution [12,19,22] and reproducible across Thailand’s remaining 75 provinces, supported by an ongoing research agenda spanning serosurveys, diagnostic validation, and post-treatment outcome studies [18,19,20,21].

Of 6719 patients evaluated for SVR12, 245 (3.65%) did not achieve cure; detailed comparison was possible for 146 of these patients against 298 patients who achieved SVR (Table 4). Treatment failure was significantly associated with male sex, any comorbidity, history of cirrhosis, injecting drug use, and irregular medication adherence (all *p* < 0.05), while age, BMI, antiviral regimen, and baseline fibrosis severity by APRI or FIB-4 did not differ significantly between groups. The absence of a fibrosis-severity gradient is notable: it suggests that, within this real-world cohort, behavioral and adherence-related factors were more strongly linked to treatment failure than baseline liver disease severity, reinforcing that programmatic success depends as much on sustained engagement through the treatment course as on clinical risk stratification at enrolment. The association with injecting drug use may reflect both nonadherence and a risk of reinfection that cannot be distinguished from true relapse without sequence-based analysis, an important area for future study. Taken together, these findings support incorporating adherence-support interventions, peer-led engagement for people who inject drugs and incarcerated individuals, integration with hemodialysis and HIV services, and selective directly observed therapy into future program iterations [54,55,57,58,59,60].

The Phetchabun model has several notable strengths. It reached all 11 districts and nearly 390,000 people across the province, surpassing WHO’s target of diagnosing 90% and treating 80%, at a scale rarely reported outside of national-level programs. The workflow was fully integrated into Thailand’s existing UHC reimbursement system and domestically financed, without reliance on external donor support, demonstrating that geographic micro-elimination is achievable using routine health-system infrastructure alone. Task-shifting of DAA prescribing to trained general practitioners at district hospitals, combined with a low-cost point-of-care RDT-to-qualitative-RNA workflow, kept the model operationally simple and reproducible. Real-time integration of screening, confirmatory testing, and treatment data into the NHSO national database further supports transparency, monitoring, and direct translation of provincial evidence into national policy, providing a template that is readily transferable to Thailand’s remaining 75 provinces and to other middle-income settings in Southeast Asia.

Several limitations of the Phetchabun model warrant explicit acknowledgment. First, internal and cross-border population mobility, including migrant workers from neighboring countries, can attenuate the long-term impact of province-limited strategies if screening and treatment are not coordinated beyond provincial boundaries, as also documented in the Catalan and Dutch experiences [60,61]. Second, residual transmission may persist in PWID, prisoners, hemodialysis patients, and PLHIV, populations that the Australian and Spanish data identify as priority targets even in low-prevalence settings [54,55,58,60]. Third, although the Thai NHSO criteria for SOF/VEL were liberalized in 2022 (removal of viral-load thresholds, relaxation of alcohol/substance-use restrictions, permissive HIV co-infection rules), historical eligibility had prioritized advanced fibrosis, potentially delaying treatment for earlier-stage patients [12]. Fourth, sustained political prioritization, stable financing, and active community engagement remain essential; the Pakistani experience emphasizes that sub-national successes can stall in the absence of central coordination and continuity [49,50,51]. Fifth, the analysis of factors associated with treatment failure (Table 4) was restricted to a sub-cohort of 444 patients with detailed case-record data available under the sub-study protocol, representing only a small fraction (5.0%) of the 8842 patients who received DAA treatment province-wide. As this sub-cohort was not randomly selected and may differ systematically from the broader treated population, these findings may not be fully representative of the entire cohort and should be interpreted with caution; moreover, only univariable (bivariate) analyses were performed, with no multivariable analysis conducted to identify independent predictors of treatment failure, so the reported associations may be subject to residual confounding and should not be interpreted as evidence of independent risk factors. This sub-cohort also did not include HIV/HBV coinfection status. Corresponding analyses of confirmatory-testing completion, treatment uptake, and loss to follow-up were not possible, as only aggregate programmatic data were available for these outcomes. Finally, long-term sero-surveillance, formal cost-effectiveness analyses, and reinfection surveillance are planned to confirm sustained impact and to support nationwide scale-up.

Global HCV micro-elimination experience converges on a consistent set of success factors: political commitment, free-of-charge testing and treatment, simplified workflows with pangenotypic DAAs, task-shifting to non-specialists, integration into existing health-system and data infrastructure, active case-finding in defined populations or geographies, and robust cascade-of-care monitoring [29,34,36,38,50,56,66].

## 5. Conclusions

The Phetchabun model contributes to this evidence base a province-wide, UHC-integrated, domestically financed, and simplified test-to-treat strategy that is directly reproducible across the remaining 75 Thai provinces and, with modest adaptation, across LMICs in Southeast Asia and beyond. Its alignment with Thailand’s existing health-system architecture, demonstrated provincial feasibility, successful policy uptake by the Department of Disease Control and NHSO, and explicitly planned expansion of test-to-treat coverage to all HCV-active adults aged 18–70 years suggests that the Phetchabun model is not merely a local achievement but a replicable reference model for accelerating Thailand’s progress toward the WHO 2030 hepatitis C elimination targets.

## Figures and Tables

**Table 1:** Cumulative HCV screening coverage and anti-HCV reactivity by district, Phetchabun province, 2020 to 30 April 2026.

No.	District	Target Population	Number Screened	%	Anti-HCV Positive	%
1	Bueng Sam Phan	26,784	28,526	106.50	392	1.37
2	Nam Nao	7672	10,417	135.78	997	9.57
3	Wang Pong	13,196	14,210	107.68	411	2.89
4	Si Thep	26,606	25,312	95.14	196	0.77
5	Lom Sak	71,518	68,866	96.29	6078	8.83
6	Chon Daen	26,957	25,943	96.24	435	1.68
7	Wichian Buri	52,579	53,049	100.89	829	1.56
8	Nong Phai	40,689	38,040	93.49	2383	6.26
9	Lom Kao	30,617	29,509	96.38	2469	8.37
10	Mueang Phetchabun	90,404	81,373	90.01	3761	4.62
11	Khao Kho	13,545	14,302	105.59	389	2.72
	Total	400,567	389,547	97.25	18,340	4.71

Note: In several districts, the number screened exceeded the registered target population because of the inclusion of unregistered residents from the official civil registration database and inward-migrant workers. Duplicate records were removed during data cleaning.

**Table 2:** Cumulative HCV-RNA confirmatory testing and HCV-RNA positivity by district, Phetchabun province.

No.	District	Anti-HCV Positive	Confirmatory HCV-RNA Tests	%	HCV-RNA Positive	%
1	Bueng Sam Phan	392	338	85.22	313	93.71
2	Nam Nao	997	964	96.69	622	62.55
3	Wang Pong	411	314	76.40	251	80.39
4	Si Thep	196	179	91.33	153	85.47
5	Lom Sak	6078	5306	87.30	4390	82.42
6	Chon Daen	435	353	81.15	209	60.83
7	Wichian Buri	829	791	95.42	510	64.05
8	Nong Phai	2383	1673	70.21	1334	79.74
9	Lom Kao	2469	2131	86.31	1439	66.99
10	Mueang Phetchabun	3761	2592	68.92	1611	60.88
11	Khao Kho	389	204	52.44	164	80.79
	Total	18,340	14,845	80.94	10,996	74.07

**Table 3:** Treatment cascade for HCV-RNA-confirmed individuals by service unit and district, Phetchabun province.

District	* Mueang Phetchabun	* Wichian Buri	* Lom Sak	Lom Kao	Nong Phai	Nam Nao	Bueng Sam Phan	Khao Kho	Chon Daen	Si Thep	Wang Pong	Total
Total Confirmed Positive (from screening)	1611	510	4390	1439	1334	622	313	164	209	153	251	10,996
Refused treatment	2	0	10	32	5	6	3	1	0	9	7	75
Lost to follow-up	21	25	0	0	7	0	1	13	0	0	3	70
Contraindications to treatment	30	0	259	10	68	0	12	4	0	0	24	407
HCC (Hepatocellular carcinoma)	26	18	14	34	46	0	14	3	8	5	13	181
Died before treatment	7	15	193	60	18	19	2	4	1	7	13	339
Treatment outside the network	174	40	242	0	76	415	0	0	8	1	7	963
Total received treatment	1696	689	3531	1303	966	152	83	105	111	52	154	8842
Treatment coverage (%)	105.28	135.10	80.43	90.55	72.41	24.44	26.52	64.02	53.11	33.99	61.35	80.4
Patients awaiting treatment	0	0	119	0	0	0	0	0	0	0	0	119

* Phetchabun Hospital serves as the central hub for the middle zone, responsible for patients in Mueang District and receiving referrals from Wang Pong Hospital, Nong Phai Hospital, Chon Daen Hospital, Khao Kho Hospital, Lom Kao Crown Prince Hospital, and Nam Nao Hospital. Lom Sak Hospital is responsible for patients within its own catchment area. Wichian Buri Hospital serves as the main hub for the southern zone, responsible for patients in Wichian Buri District and receiving referrals from Bueng Sam Phan Hospital and Si Thep Hospital.

**Table 4:** Baseline demographic and clinical characteristics of patients by direct-acting antiviral (DAA) treatment outcome (*n* = 444).

Characteristic	SVR	Treatment Failure	*p*-Value
**Participants, *n***	298	146	
**Sex, *n* (%)**			**0.002**
Male	220 (73.8)	130 (89.0)	
Female	69 (23.2)	16 (11.0)	
Age (years), mean ± SD	61.0 ± 9.5	61.1 ± 8.9	0.736
BMI (kg/m^2^), mean ± SD	23.5 ± 4.4	24.2 ± 4.3	0.082
**Comorbidities, *n* (%)**			
None	161 (54.0)	57 (39.0)	**0.004**
Diabetes mellitus, hypertension, or dyslipidaemia	134 (45.0)	71 (48.6)	0.531
Liver cirrhosis	18 (6.0)	28 (19.2)	**<0.001**
Chronic kidney disease	8 (2.7)	4 (2.7)	1.000
History of substance abuse (injection)	3 (1.0)	12 (8.2)	**<0.001**
**Medication adherence, *n* (%)**			**<0.001**
Regular adherence	283 (95.0)	118 (80.8)	
Irregular adherence	9 (3.0)	25 (17.1)	
**Antiviral regimen, *n* (%)**			0.144
Sofosbuvir/velpatasvir	261 (87.6)	136 (93.2)	
Sofosbuvir/velpatasvir + ribavirin	35 (11.7)	10 (6.8)	
**APRI score, *n* (%)**			0.286
<0.5	163 (55.4)	76 (53.1)	
0.5–1.5	101 (34.4)	45 (31.5)	
>1.5	30 (10.2)	22 (15.4)	
**FIB-4 score, *n* (%)**			0.869
<1.45	82 (27.8)	28 (29.8)	
1.45–2.67	117 (39.7)	38 (40.4)	
>2.67	96 (32.5)	28 (29.8)	
**FIB-4 in patients aged < 65 years, *n* (%)**			0.506
<1.45	71 (38.4)	26 (39.4)	
1.45–2.67	65 (35.1)	27 (40.9)	
>2.67	49 (26.5)	13 (19.7)	
**FIB-4 in patients aged ≥ 65 years (age-adjusted cut-off), *n* (%)**			0.445
<2.0	35 (32.1)	9 (32.1)	
2.0–2.67	27 (24.8)	4 (14.3)	
>2.67	47 (43.1)	15 (53.6)	

Note: Values are presented as numbers (percentages) unless otherwise indicated. Age and BMI are presented as mean ± standard deviation (SD). Categorical variables were compared between groups using the chi-square or Fisher exact test, as appropriate; continuous variables were compared using the Mann-Whitney U test. The four comorbidity categories are not mutually exclusive (a patient may have more than one); “None” indicates the absence of all four. Bold text in the Characteristic column indicates category headings; bold *p*-values indicate statistical significance (*p* < 0.05). Abbreviations: SVR, sustained virological response; BMI, body mass index; APRI, aspartate aminotransferase-to-platelet ratio index; FIB-4, fibrosis-4 index; DAA, direct-acting antiviral; SD, standard deviation.

## Data Availability

The data presented in this study are not publicly available due to ethical restrictions protecting patient privacy but are available from the corresponding author upon reasonable request.
